# Metabolic Syndrome, Clusterin and Elafin in Patients with Psoriasis Vulgaris

**DOI:** 10.3390/ijms21165617

**Published:** 2020-08-05

**Authors:** Drahomira Holmannova, Pavel Borsky, Lenka Borska, Ctirad Andrys, Kvetoslava Hamakova, Vit Rehacek, Tereza Svadlakova, Andrea Malkova, Martin Beranek, Vladimir Palicka, Jan Krejsek, Zdenek Fiala

**Affiliations:** 1 Institute of Hygiene and Preventive Medicine, Faculty of Medicine in Hradec Kralove, Charles University, 50038 Hradec Kralove, Czech Republic; holmd9ar@lfhk.cuni.cz (D.H.); svadlakovat@lfhk.cuni.cz (T.S.); MALKA8AR@lfhk.cuni.cz (A.M.); Beranek@lfhk.cuni.cz (M.B.); Fiala@lfhk.cuni.cz (Z.F.); 2 Institute of Pathological Physiology, Faculty of Medicine in Hradec Kralove, Charles University, 50003 Hradec Kralove, Czech Republic; borka@lfhk.cuni.cz; 3 Institute of Clinical Immunology and Allergology, University Hospital and Faculty of Medicine in Hradec Kralove, Charles University, 50005 Hradec Kralove, Czech Republic; andrys@fnhk.cz (C.A.); KrejsekJ@lfhk.cuni.cz (J.K.); 4 Clinic of Dermatology and Venereology, University Hospital Hradec Kralove, 50005 Hradec Králové, Czech Republic; kveta@hamakova.cz; 5 Transfusion Center, University Hospital, 50005 Hradec Kralove, Czech Republic; RehacekV@lfhk.cuni.cz; 6 Institute of Clinical Biochemistry and Diagnostics, University Hospital Hradec Kralove and Faculty of Medicine in Hradec Kralove, Charles University, 50005 Hradec Kralove, Czech Republic; Palicka@lfhk.cuni.cz

**Keywords:** psoriasis, metabolic syndrome, clusterin, elafin

## Abstract

Background: Psoriasis is a pathological condition characterized by immune system dysfunction and inflammation. Patients with psoriasis are more likely to develop a wide range of disorders associated with inflammation. Serum levels of various substances and their combinations have been associated with the presence of the disease (psoriasis) and have shown the potential to reflect its activity. The aim of the present study is to contribute to the elucidation of pathophysiological links between psoriasis, its pro-inflammatory comorbidity metabolic syndrome (MetS), and the expression of clusterin and elafin, which are reflected in the pathophysiological “portfolio” of both diseases. Material and methods: Clinical examinations (PASI score), ELISA (clusterin, elafin), and biochemical analyses (parameters of MetS) were performed. Results: We found that patients with psoriasis were more often afflicted by MetS, compared to the healthy controls. Clusterin and elafin levels were higher in the patients than in the controls but did not correlate to the severity of psoriasis. Conclusion: Our data suggest that patients with psoriasis are more susceptible to developing other systemic inflammatory diseases, such as MetS. The levels of clusterin and elafin, which are tightly linked to inflammation, were significantly increased in the patients, compared to the controls, but the presence of MetS in patients did not further increase these levels.

## 1. Introduction

Psoriasis is a systemic, chronic, and immune-mediated disease with an immunogenetic basis which can be triggered extrinsically and intrinsically by various factors (e.g., stress, mechanical trauma, infection, medication, nutrition, tobacco smoking, or obesity). Psoriasis primarily affects the skin and joints.

The hallmark of psoriasis is immune system dysfunction/overactivity leading to sustained inflammation, which is associated with keratinocyte hyperproliferation, dysfunctional differentiation, and skin barrier disruption. The most common clinical presentation of psoriasis is skin plaques with silvery scale; however, the associated inflammation has also profound pathogenic systemic effects [1,2].

Psoriasis is closely related to other (pro)inflammatory conditions, such as cardiovascular disorders, diabetes mellitus, and metabolic syndrome (MetS), which is defined as a pathological condition accompanied by abdominal obesity, dyslipidemia, insulin resistance, and hypertension [3,4]. MetS is an important risk contributor to multiple chronic diseases, such as cardiovascular diseases and several types of cancer, renal, or immunopathological disorders [5,6]. A wide range of molecules are known to be involved in the pathogenesis of psoriasis and metabolic syndrome, either as factors that drive pathogenesis or as protective factors. Two such important factors are clusterin and elafin [7,8,9,10].

Clusterin (apolipoprotein J) is a pleiotropic glycoprotein consisting of two chains—α and β—which are linked by disulfide bonds. It is synthesized in most mammalian cells, including cancer cells, as a consequence of cellular stress or TGF-β stimulation [11,12]. Clusterin may be either retained intracellularly (localized in the cytosol or in its alternatively spliced form in the nucleus) or secreted into the extracellular space and biological fluids. Several roles have been ascribed to clusterin. Intracellularly, it removes misfolded proteins and controls DNA repair. Its non-glycosylated form in the nucleus has a proapoptotic effect, but its cytosolic form inhibits apoptosis by interaction with mitochondrial Bax and reduces mitochondrial fission and ROS production [13,14]. Extracellularly, clusterin facilitates the clearance of denatured proteins, protein aggregates, and cell debris; promotes amyloid β degradation and prevents amyloid aggregation; stimulates cell proliferation; and regulates immune system activity. Clusterin shows both anti- and pro-inflammatory effects. It stabilizes I-κB and inhibits nuclear NF-κB translocation, restricts complement system activation, induces expression of TNF-α, and facilitates chemoattraction of macrophages through the activation of ERK, JNK, and PI3K/Akt pathways [15,16,17]. Importantly, clusterin has been shown to play a protective role in metabolic disorders. It modulates leptin activity, has anorexigenic properties, interacts with ghrelin, decreases the activity of SREBP-1C (a regulator of several lipid pathways), and reduces hepatic lipid accumulation [18,19,20].

Elafin, also known as SKALP (skin-derived anti-leukoprotease), is a serine protease inhibitor belonging to the chelonians, which are released from pre-elafin by proteolytic cleavage, that may be mediated by mast cell-derived tryptase [21,22]. The main sources of elafin are epithelial cells and keratinocytes. In normal skin, elafin is almost undetectable; however, its level is increased in psoriatic lesions, reflecting their severity. The expression is enhanced by IL-1 and TNF-α and, thus, by pro-inflammatory microenvironments [23,24]. Elafin can inhibit neutrophil serine protease, neutrophil elastase, proteinase 3, and endogenous vascular elastase. Interestingly, elafin is cleaved by its target neutrophil elastase [25,26]. Elafin protects tissues against the damage caused by pathological acute and chronic inflammation (e.g., infections, graft rejection, colitis, diabetes, and inflammatory skin diseases). Chronic inflammation is commonly associated with increased neutrophil-released enzyme activity, elafin suppresses the immune response and exhibits antimicrobial effects against different classes of infectious pathogens (i.e., viruses, bacteria, parasites, and fungi) [27,28].

Considering the above, psoriasis and its comorbidities are associated with very complex pathophysiological mechanisms. The aim of the present study is to contribute to the elucidation of the pathophysiological links between psoriasis, its pro-inflammatory comorbidity MetS, and the expression of selected factors (clusterin and elafin) which are reflected in the pathophysiological “portfolio” of both diseases (psoriasis and MetS).

## 2. Methods

### 2.1. Study Groups

The experimental group consisted of 45 patients with psoriasis. Patients were investigated at the Clinic of Dermal and Venereal Disease, Charles University Hospital in Hradec Kralove. Patients with inflammatory diseases (e.g., infectious diseases, malignancy, or inflammatory rheumatic diseases), those who were pregnant, and those using non-steroidal or anti-inflammatory medications were excluded from the study. Patients with psoriasis did not have any form of psoriasis treatment three months before the study. The control group included 40 healthy blood donors from the Department of Transfusion Medicine, Charles University Hospital in Hradec Kralove.

All subjects gave their informed consent for inclusion before they participated in the study. The study was conducted in accordance with the Declaration of Helsinki, and the protocol was approved by the Ethics Committee of the Charles University Hospital in Hradec Kralove, Czech Republic (Project identification code PROGRES Q40-09 and Q40-10, reference number 201705 I83P, date 2 May 2017).

### 2.2. PASI Score

The severity of the disease was assessed using a standardized clinical evaluation, the Psoriasis Area Severity Index (PASI score), which is calculated based on erythema, desquamation, and skin infiltration [29].

### 2.3. Blood Samples Collection

Peripheral blood samples were collected from the cubital vein of all persons in both groups using BD Vacutainer sampling tubes. Blood serum samples were isolated by centrifugation and stored at −70 °C until analysis. Repeated thawing and freezing were avoided.

### 2.4. Metabolic Syndrome (MetS) and BMI

Evaluation of the presence of MetS in observed subjects was carried out in accordance with the criteria of the National Cholesterol Education Program Adult Treatment Panel (NCE/ATPIII). The diagnosis of MetS can be declared when three of the five following listed criteria are present: increased waist circumference or abdominal obesity (≥102 cm for men and ≥88 cm for women), glucose intolerance presented by higher fasting glucose (≥5.6 mmol/L) or known treatment for diabetes, raised level of triglycerides (TAG; ≥1.7 mmol/L) and reduced level of high-density lipoproteins (HDL cholesterol), and elevated blood pressure (systolic blood pressure, SBP ≥ 130 mmHg, and/or diastolic blood pressure, DBP ≥ 85 mmHg) [4,30]. Body mass index (BMI) was calculated as the ratio of weight to height squared (kg/m^2^).

### 2.5. Analysis of Clusterin

Serum levels of clusterin were determined using the ELISA kit Quantikine Human Clusterin (R&D Systems, MN, USA), according to the manufacturer’s instructions. The minimum detectable dose of human Clusterin ranged from 0.064–1.050 ng/mL. The samples were 2000-fold diluted. The absorbance values were read at 450 nm on a Multiskan RC ELISA reader (Thermo Fisher Scientific, Waltham, MA, USA).

### 2.6. Analysis of Elafin

Serum levels of Elafin were determined using the ELISA kit Elafin/Skalp Human ELISA Kit (Abcam, UK), according to the manufacturer’s instructions. The sensitivity of the kit was 5 ng/mL. Samples were 150-fold diluted. The absorbance values were read at 450 nm on a Multiskan RC ELISA reader (Thermo Fisher Scientific, Waltham, MA, USA).

### 2.7. Statistical Analysis

The data were statistically processed with the Statistica software version 13.5.0.17 (TIBCO Software Inc., Palo Alto, CA 94304 USA). We used both parametric and nonparametric tests, based on the Shapiro–Wilk test for the data distribution, in order to ensure proper test sensitivity. Intergroup differences were assessed using the Student’s *t*-test or the Mann–Whitney *U*-test. The differences were considered statistically significant when the probability level (*p*) was below the alpha level of 0.05. Associations between the parameters were evaluated by the Spearman’s rank correlation test.

## 3. Results

### 3.1. Participants Data

**Figure 1:**
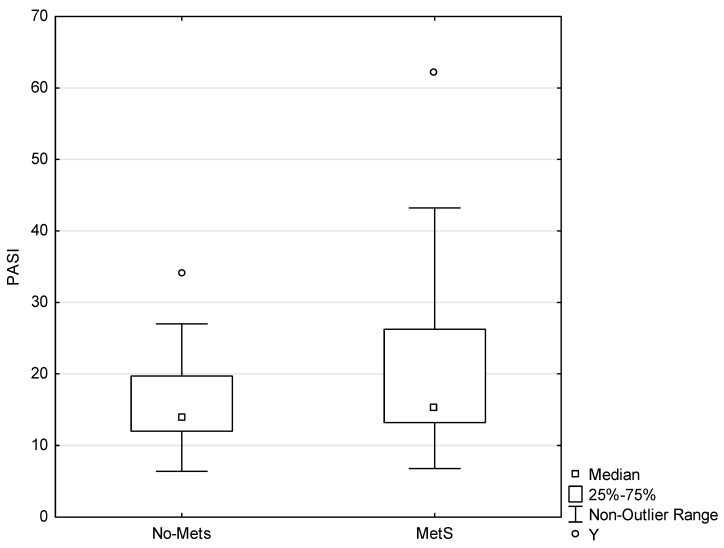
Psoriasis Area Severity Index (PASI) score difference between the MetS patients and the no-MetS patients. Y indicates an outlier. An outlier is any data point value >75th percentile + 1.5 * (75th percentile–25th percentile) or any data point <25th percentile–1.5 * (75th percentile–25th percentile), *p* < 0.05.

A total of 85 subjects were enrolled in the study. The age distribution did not differ significantly between the group of patients and the controls (Table 1). The study included 24 patients with MetS (MetS patients), 21 patients without MetS (no-MetS patients), 7 controls with MetS (controls MetS), and 33 controls without MetS (controls no-MetS). The prevalence of MetS was significantly higher among the patients (*p* < 0.01). There was no significant difference in the level of PASI score between the MetS patients and the no-MetS patients (*p* = 0.3567; Figure 1).

### 3.2. Clusterin Analysis

**Figure 2:**
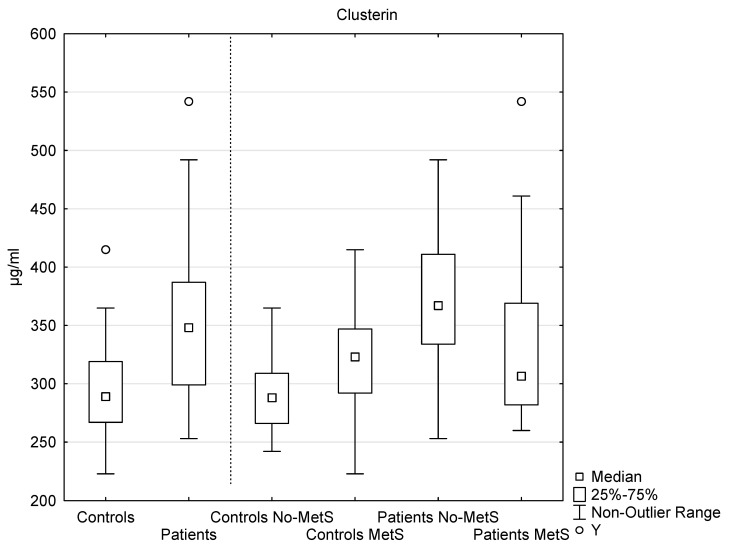
The levels of serum clusterin in the controls and in the patients, as well as the levels of serum clusterin in the subgroups of the controls and the patients with and without metabolic syndrome. Y indicates an outlier. An outlier is any data point value >75th percentile + 1.5 * (75th percentile–25th percentile) or any data point <25th percentile–1.5 * (75th percentile–25th percentile). Controls vs. patients, *p* < 0.001; MetS patients vs. no-MetS, *p* < 0.05; no-MetS patients vs. no-MetS control, *p* < 0.001.

The serum level of clusterin was significantly elevated in the patients, compared to the controls: patients (*n* = 45, median 348 µg/mL, interquartile range 299–387 µg/mL); controls (*n* = 40, median 289 µg/mL, interquartile range 267–319 µg/mL); *p* < 0.001 (Figure 2).

In the MetS patients (*n* = 24, median 307 µg/mL, interquartile range 282–369 µg/mL), the level of clusterin was significantly lower than in the no-MetS patients (*n* = 21, median 367 µg/mL, interquartile range 334–411 µg/mL), *p* < 0.05. In the no-MetS patients, the level was significantly elevated, in comparison with the no-MetS controls (*n* = 33, median 288 µg/mL, interquartile range 266–309 µg/mL), *p* < 0.001 (Figure 2).

### 3.3. Elafin Analysis

**Figure 3:**
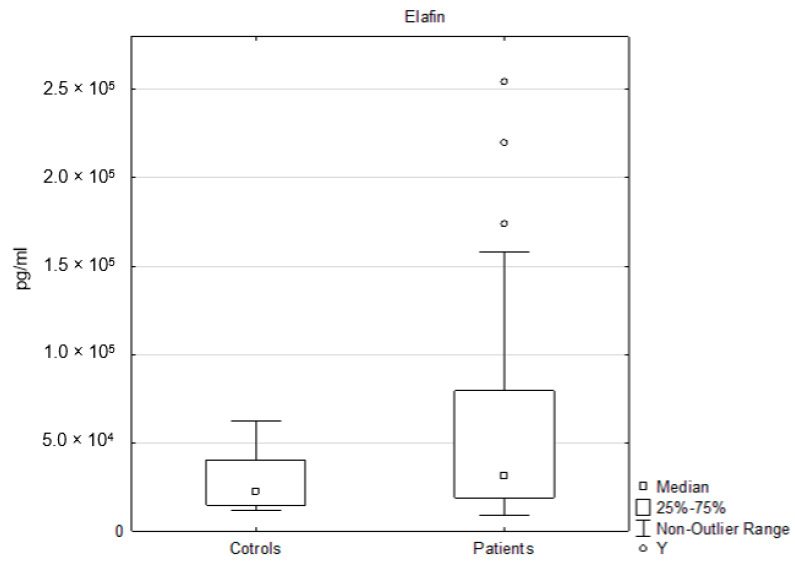
The levels of serum elafin in the controls and in the patients. Y indicates an outlier. An outlier is any data point value >75th percentile + 1.5 * (75th percentile–25th percentile) or any data point <25th percentile–1.5 * (75th percentile–25th percentile), *p* < 0.05.

The serum level of elafin was significantly elevated in the patients, compared to the controls: patients (*n* = 45, median 30,900 pg/mL, interquartile range 18,800–79,400 pg/mL); controls (*n* = 40, median 22,250 pg/mL, interquartile range 14,700–40,400 pg/mL); *p* < 0.05 (Figure 3).

### 3.4. Relationships Between the Evaluated Parameters

In both observed groups (patients and controls), we tested the possible relationships between (1) clusterin (elafin) and age; (2) clusterin (elafin) and BMI; (3) clusterin (elafin) and PASI score; (4) clusterin (elafin) and glucose, triglycerols, HDL cholesterol, and waist circumference; and (5) between clusterin and elafin. The only tested relationship that reached the level of statistical significance was between clusterin and waist circumference in patients (*r* = −0.409; *p* < 0.01). In the group of controls, the relationship between elafin and age approached the limit of statistical significance (*r* = 0.30; *p* = 0.059).

## 4. Discussion

Psoriasis is a multifactorial systemic immune-mediated disease with global incidence and prevalence increasing over recent decades. There is evidence that persons with psoriasis have a higher risk of developing other chronic (pro)inflammatory medical conditions, including metabolic syndrome, which directly promotes the development of cardiovascular disease and diabetes mellitus type II. In agreement with the findings of other authors, the prevalence of metabolic syndrome among participants in our study was higher among the patients with psoriasis, compared to the healthy controls [31,32,33].

The presence and progression of psoriasis is mirrored by the changes in expression of various molecules which have the potential to (down/up) regulate the inflammatory activity of the immune system and, thus, the activity of the disease; however, not all of them are highly representative of psoriasis and do not provide valuable information regarding psoriasis. Finding biomarkers that provide a link to psoriasis and its severity is, therefore, important [1]. We analyzed the expression of two potential markers of psoriasis: clusterin and elafin. The expression of both molecules depends on the presence of an ongoing inflammatory response.

There is a very limited number of studies that have analyzed the level of clusterin in patients with psoriasis. In our study, the serum level of clusterin was significantly elevated in the patients, compared to the controls. The same result was obtained in the study performed by Buquicchio et al., where the level of clusterin in 15 patients was higher than in the 10 controls [34]. Ataseven et al. did not detect a significant difference between the levels of clusterin in patients and controls [35]. On the other hand, García-Rodríguez et al. documented that the level of clusterin was reduced in the patients, compared to the control group [36]. Thus, conflicting results exist. Clusterin may serve as either a protective or aggravating factor in the pathogenesis of psoriasis.

We assumed that clusterin might play a protective role (as a compensatory mechanism) in psoriasis, metabolic syndrome, and other inflammatory (e.g., rheumatoid arthritis and atopic dermatitis) and neurodegenerative (e.g., Alzheimer’s and Parkinson’s) diseases. These diseases have been shown to be accompanied by an overexpression of clusterin, which displays anti-inflammatory properties [37,38,39].

Psoriasis, as a systemic inflammatory condition, leads to the activation of the complement cascade with MAC formation (membrane attack complex; Cb5-9) and the increased expression of metalloproteinases [40]. Importantly, clusterin binds to MAC and prevents cytolysis and tissue injury [17]. It also modulates the inflammatory activity of dendritic cells which are also involved in the progression of psoriasis; especially subset CD11c^+^ mDCs [41].

Clusterin deficiency in mice intensified the allergic inflammation and infiltration of airways by dendritic cells [42]. In a mouse model of acute pancreatitis, clusterin-deficient mice showed a more severe course of the disease. The pancreatic tissue was massively infiltrated by inflammatory cells, and the rate of apoptosis was enhanced; on the contrary, the presence of clusterin inhibited the activity of NF-κB, which regulates the expression of genes encoding TNF-α. Furthermore, clusterin has shown anti-inflammatory and cytoprotective activity [43].

We cannot omit the chaperone functions of clusterin. Inflammation and oxidative stress (the conditions that accompany psoriasis) contribute to cell death (i.e., apoptosis and necrosis), the formation of cell debris, the unfolding of extracellular proteins, and their aggregation. These processes and changes might create new epitopes to which the immune system is intolerant, and autoimmunity emerges when self-tolerance mechanisms fail. Thus, it is extremely important to clear these dangerous factors. Clusterin, as an extracellular chaperon, exhibits the characteristics of innate immune system receptors and facilitates such clearance, thus providing protection against the development of autoimmune disease [44].

Interestingly, Th17 cells (a subset of CD4^+^ T cells), which play a crucial role in pathogenesis of psoriasis, exhibit a lower expression of clusterin and a higher expression of Bcl-2, which make them less susceptible to apoptosis [45].

The subgroup analysis of clusterin levels revealed that the presence of psoriasis (inflammation and oxidative stress) significantly elevated the level of clusterin; the level of clusterin was also higher in the no-MetS patients compared to the no-Mets controls. We also detected a significant difference between the clusterin levels in the MetS patients and the no-MetS patients. Surprisingly, the level of clusterin was higher in no-MetS patients. Although both psoriasis and metabolic syndrome were associated with the elevation of clusterin levels, the presence of the combination of diseases did not elevate the expression of clusterin, compared to psoriasis alone. The lower level of clusterin in the MetS patients, compared to the no-MetS, might be caused by a wide variety of mechanisms.

Clusterin is involved not only in inflammatory reactions but also in energy and lipid metabolism and forms complexes with lipids, leptin, and proteins [46]. These complexes interact with receptors (megalin, ApoER2, VLDL R), which are internalized and degraded in lysosomes or proteasomes (inhibition of lysosome/proteasome results in the accumulation of intracellular clusterin without altering mRNA expression) [47]. It has been also described that the clusterin accumulates in the walls of atherosclerotic arteries [48].

Endoplasmic reticulum (ER) stress also plays a role, which may be induced by inflammation and hyperlipidaemia. Normally, clusterin is translocated from the ER into the Golgi apparatus and released from the cell. Under stress conditions, the re-translocation of clusterin to cytosol or mitochondria has been documented [49]. This results in a decline in the expression of serum clusterin, although the expression of the gene for clusterin is upregulated. Re-translocation and accumulation of clusterin intracellularly depends on the ER-associated ubiquitin ligase HRD1 and chaperone GRP78. It is well-known that HRD1 controls body weight. Its depletion/suppression protected against obesity, hyperlipidaemia, and insulin resistance in mouse models; moreover, its expression was more elevated in obese mice, compared to the controls [50,51,52]. The expression of HRD1 is regulated by NFE2L1 (nuclear factor erythroid 2-related factor-1), which plays an important role in lipid homeostasis and directly binds cholesterol in ER and activates genes responsible for cholesterol excretion. It is a key component of the adaptive response to excess cholesterol in cells [53,54].

Similarly to HRD1, GRP78 controls the stability, re-translocation, and mitochondrial localization of clusterin. Shimoura et al. developed a mouse model of psoriasiform inflammation, either with obesity and diabetes or without obesity and diabetes. The expression of GRP78 was significantly increased in the skin of diabetic and obese mice, compared to the controls [55]. Sukuira et al. noted that the expression of both GRP78 and HRD1 was markedly enhanced in keratinocytes undergoing differentiation from normal human skin but was significantly decreased in proliferating keratinocytes in psoriasis vulgaris lesions [56].

In summary, clusterin might form complexes, be more intensively endocytosed, and be redistributed in patients with MetS, which implies that the level of clusterin in serum in MetS patients might be decreased, compared to no-MetS patients. ER stress and the expression of factors supporting the re-translocation and degradation of clusterin are more expressed in environments associated with metabolic syndrome (e.g., hyperlipidaemia, glucose-intolerance, and obesity) than in psoriasis. It may be assumed that both metabolic syndrome and psoriasis increase the synthesis and release of clusterin, compared to normal conditions, but the factors associated with metabolic syndrome might lower the level of clusterin, compared to that in psoriasis alone.

Although clusterin is an important player in energy and lipid metabolism, we did not confirm any correlation between clusterin and BMI, either in the patients or in the controls. In the study of Arnold et al., the same results were observed: Baseline clusterin did not differ between lean and obese participants. The level of clusterin decreased during weight reduction, but the decrease did not correlate with BMI. Thus, the authors suggested that changes in clusterin concentration depended on the reduction in caloric intake, not on the weight loss [57]. Similarly, Klouckova et al. confirmed that the plasma levels of clusterin did not differ between obese patients and healthy controls but were influenced by low caloric intake [58].

Clusterin might play a protective role and prevent tissue damage. Its higher level in patients with psoriasis and metabolic syndrome may represent a compensatory protective mechanism.

Besides clusterin, we measured the levels of elafin, which were significantly elevated in the patients, compared to the controls. The results presented by Elgharib et al. were consistent with those of our research. The level of elafin was much higher in the patients than in the controls [23]. Alkemade et al. also confirmed that the levels of serum and urine elafin were markers of psoriasis and the activity of the disease. Cyclosporin therapy improved PASI and lowered the level of elafin; thus, the level of elafin was correlated with PASI. We did not document the relationships between elafin and PASI and BMI. An elevated level of elafin was also detected, by Wang et al., in prediabetic subjects. This increase was associated with a reduction in body fat mass and levels of fasting blood glucose, which was mediated by elafin-induced leptin production [59].

While we can only speculate whether clusterin has a protective capacity, elafin is undoubtedly a protective factor with anti-inflammatory properties. Elafin is a potent inhibitor of neutrophil elastase and other proteolytic enzymes, and its expression is enhanced in inflammatory microenvironments. Neutrophil elastase is a major component of azurophilic granules of neutrophils. Neutrophil elastase has anti-microbial activity, but it can also harm the organism. It cleaves various substrates, such as elastin, laminin-332, epithelial cadherin, surfactant proteins, C5a receptors, C1 inhibitors, VEGF, and insulin receptors; increases the production of inflammatory and chemotactic cytokines IL-8, IL-6, and TNF-α; and initiates the formation of neuroendocrine tumors (NETs). These factors intensify the inflammatory response and drive tissue damage, including microvascular injury and increased vascular permeability and epithelial disruption, preferentially in the airways and intestinal tract, as well as tissue remodeling [60,61].

Elafin attenuates autoimmune (diabetes, colitis, coeliac disease), allergic (asthma, atopic dermatitis), and chronic inflammation (atherosclerosis), as well as acute LPS-induced (lipopolysaccharides-induced) inflammation and sepsis. The anti-inflammatory and protective properties of elafin are mainly based on its ability to cleave, and, thus, inhibit the proinflammatory effect of, neutrophil elastase. In addition to its antiprotease properties, elafin reduces the activity of NF-κB (re-translocation into the nucleus). Consequently, the production of proinflammatory cytokines (e.g., TNF-α and IL-8) and epithelial damage are reduced [25,27,62,63,64,65,66].

Therefore, elafin can re-equilibrate the balance between protease and anti-protease activities, prevent and reduce inflammation, accelerate the resolution of inflammation, and protect against irreversible tissue damage.

The total number of subjects enrolled in the study (*n* = 85), as well as the number of subjects in each main group (patients: *n* = 45 and controls: *n* = 40), was satisfactory to ensure sufficient power of the study. On the other hand, the subgroup analysis might have been limited by the small sample sizes (MetS patients: *n* = 24, no-MetS: *n* = 21; MetS controls *n* = 7, no-MetS *n* = 30). ANOVA tests were not conducted, as the differences were always compared between two groups, ensuring better test sensitivity in smaller groups.

## 5. Conclusions

Our results confirmed the link between psoriasis and metabolic syndrome. This may suggest that patients with psoriasis are more prone to developing metabolic disorders than healthy persons. The levels of clusterin and elafin (which are tightly linked to inflammation) were significantly elevated in patients, in comparison with the controls. It seems that these factors play a protective role and reduce the tissue damage caused by the inflammation associated with psoriasis and metabolic syndrome.

## Figures and Tables

**Table 1:** Age, body mass index (BMI) and metabolic syndrome (MetS) in participants.

	Controls				Patients			
	*N*	Median	Q1	Q3	*N*	Median	Q1	Q3
Age	40	50.5	36.9	57.4	45	52.9	40.5	67.6
BMI *	40	24.3	23.1	27.5	45	28	24.8	30.4
Glc *	40	4.26	3.63	4.66	45	4.77	4.40	5.46
TAG	40	1.23	0.84	1.64	45	1.57	0.98	2.02
HDL *	40	1.30	1.16	1.52	45	1.08	0.91	1.28
Waist	40	88	80	99	45	98	87	103
BPsys	40	130	120	140	45	130	125	140
BPdia *	40	80	75	85	45	90	80	95

Legend: Q1 = lower quartile, Q3 = upper quartile, Glc = glucose, TAG = triacylglycerols, HDL = high density lipoproteins, Waist = waist circumference, BPsys = systolic blood pressure, BPdia = diastolic blood pressure, * significant difference between controls and patients (*p* < 0.05).
